# *Pueraria thomsonii* Radix Water Extract Alleviate Type 2 Diabetes Mellitus in db/db Mice through Comprehensive Regulation of Metabolism and Gut Microbiota

**DOI:** 10.3390/molecules28227471

**Published:** 2023-11-07

**Authors:** Jiarong Li, Hua Zhang, Hui Ouyang, Weixin Xu, Yong Sun, Youbao Zhong, Lifang Wang, Jiaxing Huang, Junchang Chen, Mingyao Li, Weifeng Zhu, Yuhui Liu, Ronghua Liu

**Affiliations:** 1 School of Pharmacy, Jiangxi University of Chinese Medicine, Nanchang 330002, China; 20131048@jxutcm.edu.cn (J.L.); 20070982@jxutcm.edu.cn (H.O.); xuweixin30@jxutcm.edu.cn (W.X.); 20171006@jxutcm.edu.cn (Y.Z.); 202001006081@jxutcm.edu.cn (L.W.); 202001006113@jxutcm.edu.cn (J.H.); 202001006201@jxutcm.edu.cn (J.C.); 202001006170@jxutcm.edu.cn (M.L.); 2 Department of Food Nutrition and Safety, College of Pharmacy, Jiangxi University of Chinese Medicine, Nanchang 330004, China; 20191002@jxutcm.edu.cn; 3 State Key Laboratory of Innovative Drug and Efficient Energy-Saving Pharmaceutical Equipment, Nanchang 330006, China; 4 State Key Laboratory of Food Science and Technology, Nanchang University, Nanchang 330047, China; yongsun@ncu.edu.cn; 5 Key Laboratory of Modern Preparation of Chinese Medicine, Jiangxi University of Chinese Medicine, Nanchang 330002, China; 19930220@jxutcm.edu.cn

**Keywords:** *Pueraria thomsonii* Radix, type 2 diabetes mellitus, metabolomics, gut microbiota

## Abstract

Type 2 diabetes mellitus (T2DM) is an increasingly prevalent and serious health problem. Its onset is typically associated with metabolic disorders and disturbances in the gut microbiota. Previous studies have reported the anti-T2DM effects of *Pueraria thomsonii* Radix as a functional food. However, the mechanism of action is still unknown. In this study, rich polyphenols and polysaccharides from *Pueraria Thomsonii* Radix water extract (PTR) were quantitatively determined, and then the effects of PTR on db/db mice were evaluated by pharmacology, metabolomics, and 16S rRNA gene sequencing. The results showed that PTR could alleviate pancreatic tissue damage, significantly decrease fasting blood glucose (FBG), fasting serum insulin (FINS), homeostasis model assessment insulin resistance (HOMA-IR), urinary glucose (UGLU), and urinary albumin/creatinine ratio (UACR). Metabolomics showed that the Diabetes Control (DM) group produced 109 differential metabolites, of which 74 could be regulated by PTR. In addition, 16S rRNA sequencing was performed in fecal samples and results showed that PTR could reduce the *Firmicutes/Bacteroidetes(F/B)* ratio and regulate three beneficial bacteria and one harmful bacterium. In conclusion, the results showed that PTR could ameliorate the T2DM symptoms, metabolic disorder, and gut microbiota imbalance of db/db mice, and it was superior to metformin in some aspects. We suggested for the first time that γ-aminobutyric acid (GABA) may be involved in the regulation of the microbiota–gut–brain axis (MGB) and thus affects the metabolic disorders associated with T2DM. This study will provide a scientific basis for the development of functional food with PTR.

## 1. Introduction

Diabetes is a complex metabolic disease associated with disorders of glucose or lipid metabolism, with chronic hyperglycemia as the main feature. The number of adults with diabetes worldwide has reached 537 million in 2021, of which type 2 diabetes mellitus (T2DM) accounts for the vast majority [1,2], and the total incidence will continue to surge with the aging of the world population. T2DM has become a major challenge for people’s health and has caused a worldwide economic burden. In the development process of T2DM, as time goes on, the clinical symptoms of “three more and one less” (food intake and water intake, fasting blood glucose, weight loss) often appear [3]. Currently, dietary intervention, intensive exercise programs, insulin, and oral hypoglycemic agents are the most common approaches to combat T2DM, while the use of medicinal and food homologous functional foods for intervention is believed to be more beneficial in alleviating T2DM, with fewer side effects. The theory of “medicine and food homology” was formally proposed in the 1920s and 1930s [4]. Medicinal and food homologous plants are often developed into functional foods due to being rich in bioactive compounds, which have health-promoting effects with minimal side effects. As a low glycemic index product, *Pueraria thomsonii* Radix has been used as food and medicine for thousands of years in Asian countries such as China, Thailand, Vietnam, and Japan. *Pueraria thomsonii* Radix is a traditional Chinese herbal medicine that can be used to treat “wasting and thirsting disorder” according to the Chinese Pharmacopoeia [5]. Furthermore, modern studies have emphasized that the bioactive components derived from *Pueraria thomsonii* Radix can help to improve fasting blood glucose in db/db mice, with limited side effects [6,7]. In previous studies, many monomer components of polyphenols extracted from *Pueraria thomsonii* Radix, such as puerarin, daidzein, and genistein, or effective parts such as polysaccharides, have been shown to regulate animal insulin resistance to control blood glucose [8,9], but there is no evidence that the effect of monomer components or effective parts is better than the overall effect of *Pueraria thomsonii* Radix [7,8,9,10,11]. Therefore, a comprehensive understanding of the comprehensive mechanism for preventing the development of T2DM in *Pueraria thomsonii* Radix still has limitations. We speculate that PTR, as a water extract of *Pueraria thomsonii* Radix, contains both polyphenols and polysaccharides, which can better represent its overall efficacy and contribute to the development of functional foods.

**Figure 1:**
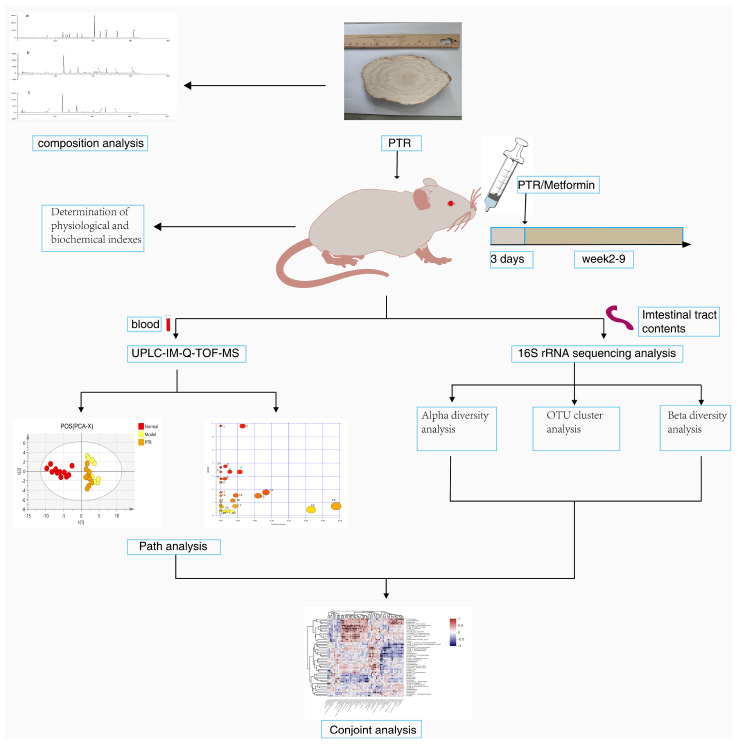
Schematic diagram of this study.

Mounting evidence has demonstrated that a combination of metabolism and gut microbiota may serve as a potentially crucial factor in T2DM diagnosis, pharmaceutical discovery, as well as therapeutic response monitoring [10,11]. In the present study, due to deficient leptin receptor genes, db/db mice can spontaneously develop obesity and chronic hyperglycemia; thus, we used db/db mice as T2DM model mice and used db/m mice as the normal control mice, then developed PTR water extract and performed pharmacological analysis after intra-gastric administration. We compared PTR with metformin in meliorating the clinical pathological feature of db/db mice, and then explored the gut microbiota and host metabolomic reactions through UPLC-IM-Q-TOF-MS [12,13] and 16S rRNA gene sequencing to understand the comprehensive antidiabetic mechanism of PTR. We further propose the potential mechanism through which PTR participates in metabolic regulation through the microbiota–gut–brain axis (MGB). Figure 1 summarizes the schematic diagram of this study.

## 2. Results

### 2.1. Chemical Composition Analysis

Quantitative analysis revealed that PTR contains nine natural polyphenols and rich total polysaccharides (Table 1). These polyphenols and polysaccharides may be the main functional components of PTR.

### 2.2. PTR Improves Major Physiological and Biochemical Indexes in db/db Mice

**Figure 2:**
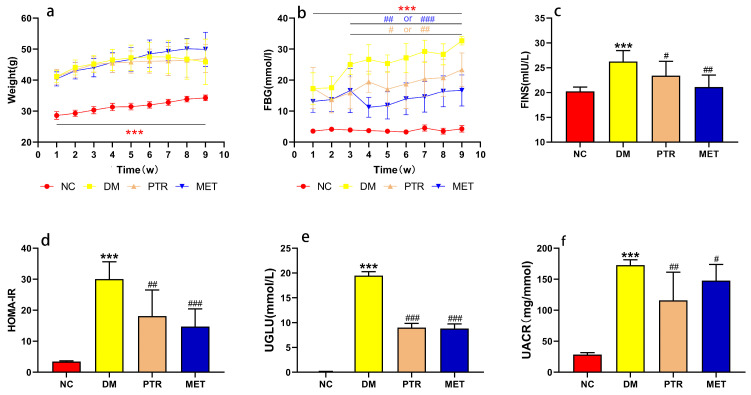
Effect on type 2 diabetes mellitus (T2DM) symptoms in db/db mice: (**a**) weight; (**b**) fasting blood glucose (FBG); (**c**) fasting serum insulin (FINS); (**d**) homeostasis model assessment insulin resistance (HOMA-IR); (**e**) urinary glucose (UGLU); (**f**) urinary albumin/creatinine ratio (UACR). Compared NC group with DM group, the significance of the difference was described as *** *p* < 0.001. Compared with DM group, the significance of the difference after the administration of PTR and metformin was described as ^#^ *p* < 0.05, ^##^ *p* < 0.01, ^###^ *p* < 0.001.

Weight, fasting blood glucose (FBG), fasting serum insulin (FINS), homeostasis model assessment insulin resistance (HOMA-IR), urinary glucose (UGLU), and urinary albumin/creatinine ratio (UACR) are commonly used indicators to evaluate the development and control level of T2DM. As shown in the experimental results in Figure 2a,b, the body weight and FBG of the DM group was significantly higher than that of the NC group in the first weekend of this study. Subsequently, there was no significant difference in the body weight of each group of db/db mice at the weekend of every week, but the DM group showed a trend of weight loss starting at the sixth weekend. Compared with the NC group, the FBG of DM groups had a rising tendency and had a significant difference compared with the NC group every weekend. However, after PTR and metformin administration, the trend in FBG elevation was alleviated, and there was a significant difference between these two groups and the DM group from the third weekend of treatment. In addition, FINS, HOMA-IR, UGLU, and UACR in the PTR and MET groups were significantly lower than those in the DM group at the end of the ninth week after euthanasia (Figure 2c–f).

### 2.3. PTR Alleviates Pancreatic Tissue Damage in db/db Mice

**Figure 3:**
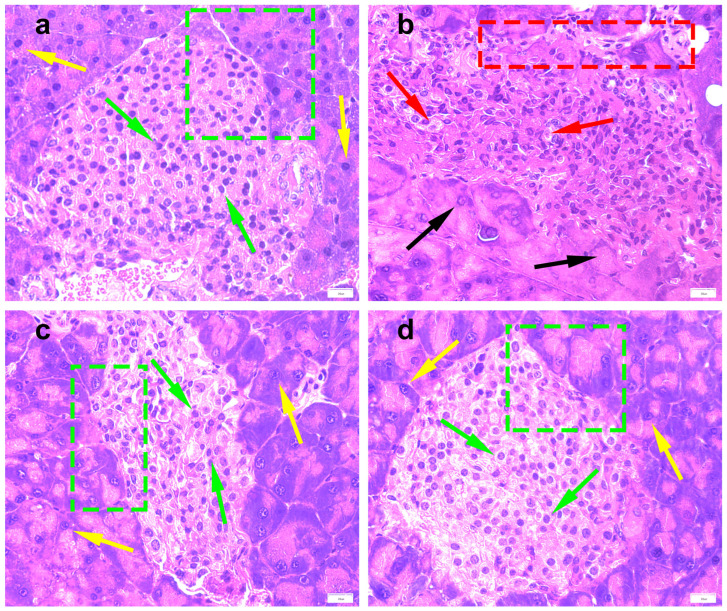
Photomicrographs of pancreas in (**a**) NC group (0); (**b**) DM group (+3); (**c**) PTR group (+1); and (**d**) MET group (0) at 400× total magnification. Based on the pathological changes in the understudied tissues, the histological sections were rated approximately on a scale from 0 to 4, with no change (0), minimal changes (+1), mild changes (+2), moderate changes (+3), and severe changes (+4). The green arrow indicated normal pancreatic islet cells, the red arrow indicated moderately swollen or hollowed out pancreatic islet cells, the yellow arrow indicated normal pancreatic acinar cells, and the black arrow indicated degenerative or necrotic pancreatic acinar cells., The red box indicated that the pancreatic islets were atrophied, and the boundary between islet cells and pancreatic acinar cells was unclear. The green box showed a clear boundary between pancreatic islet cells and pancreatic acinar cells, and the morphology of both cells was normal.

The observation of mouse pancreas HE staining showed that the NC group (Figure 3a) demonstrated normal organized morphological features of pancreatic parenchyma, with many apparent intact pancreatic islets with normal subcellular structures (green arrow) and intact pancreatic acini (yellow arrow) with intact interlobular scanty connective tissue with intact vasculatures. Moreover, the boundary between islet cells and pancreatic acinar cells was clear (green box). In the DM group (Figure 3b), the pancreatic islets were atrophied, and the boundary between islet cells and pancreatic acinar cells was unclear (red box). Moreover, a large number of islets cells demonstrated moderate swelling and vacuolar degeneration (red arrow), and a large number of pancreatic acinar cells were necrotic and degenerative (black arrow). After the administration of PTR and metformin (Figure 3c,d), two types of cells were observed with clear boundaries (green box), and an increase in the number of normal cells (green arrow and yellow arrow) was observed. This suggested that the pancreatic tissue damage of db/db mice can be potentially alleviated by the administration of PTR and metformin.

### 2.4. The Modulatory Effects of PTR on Serum Metabolites in db/db Mice

**Figure 4:**
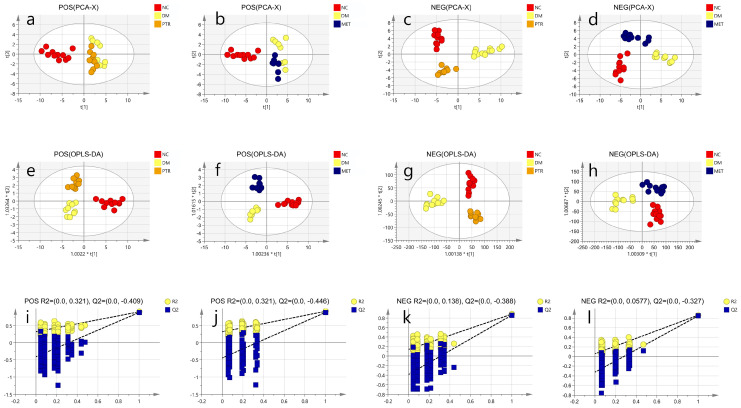
Untargeted metabolomic analysis of each group: (**a**,**b**) POS, PCA; (**c**,**d**) NEG, PCA; (**e**,**f**) POS, OPLS-DA; (**g**,**h**) NEG, OPLS-DA; (**i**,**j**) POS, R2, Q2; (**k**,**l**) NEG, R2, Q2.

**Figure 5:**
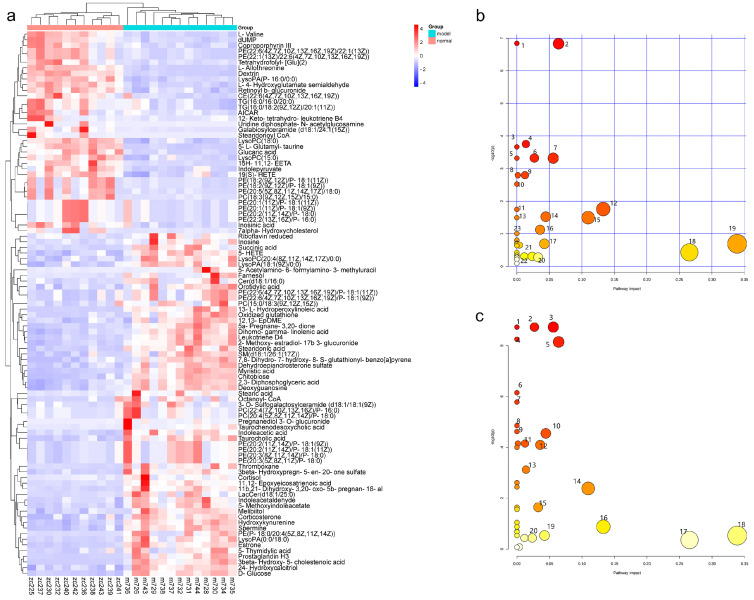
Effect on metabolomics of serum in db/db mice: (**a**) heat map analysis of potential biomarker relative content; (**b**) summary plot for pathway analysis of PTR (1–15, 18–19 metabolic pathway satisfies the conditions of *p* < 0.05 or impact value > 0.1); (**c**) summary plot for pathway analysis of metformin (1–18 metabolic pathway satisfies the conditions of *p* < 0.05 or impact value > 0.1).

A significant separation was observed among the NC group, DM group, PTR, or MET groups in the PCA and OPLS-DA score graphs. The R2 and Q2 values in OPLS-DA were smaller than the original values, indicating that the established discriminant model was reliable. The above results indicated that PTR and metformin could significantly alter the composition of serum metabolites in db/db mice (Figure 4). VIP > 1 and *p* < 0.05 are commonly used criteria for screening potential biomarkers. In this study, we screened according to the above criteria and ultimately identified 109 differential metabolites (Supplementary Table S2), mainly 13 types of substances, including lipids, lipid-like molecules, bile acids, alcohols and their derivatives, glycerol phospholipids, nucleosides, nucleotides and analogues, steroids and steroid derivatives, organic oxygen compounds, and organic heterocyclic compounds. Heat map analysis of these relative metabolites is shown in Figure 5a. The results from the analysis of the differential metabolites with callback after administration in db/db mice showed that there were 74 callback differential metabolites after the administration of PTR, and their abundance values are shown in Supplementary Table S3. In addition, there were 49 callback differential metabolites after the administration of metformin, and their abundance values are shown in Supplementary Table S4. To determine the difference between the PTR group and MET group, the concentration table of differential metabolites introduced into PTR and metformin was used to analyze the enrichment path of their differential metabolites through MetaboAnalyst Path analysis and KEGG (https://www.metaboanalyst.ca/)(accessed on 18 October 2022). Finally, 40 metabolic pathways (Supplementary Table S5) were particularly enriched by PTR, and 26 metabolic pathways were enriched by metformin (Supplementary Table S6). There were 24 main metabolic pathways overlapped between PTR and metformin, as shown in the Bubble Diagram (Figure 5b,c).

### 2.5. PTR Improved the Gut Microbiota in db/db Mice

**Figure 6:**
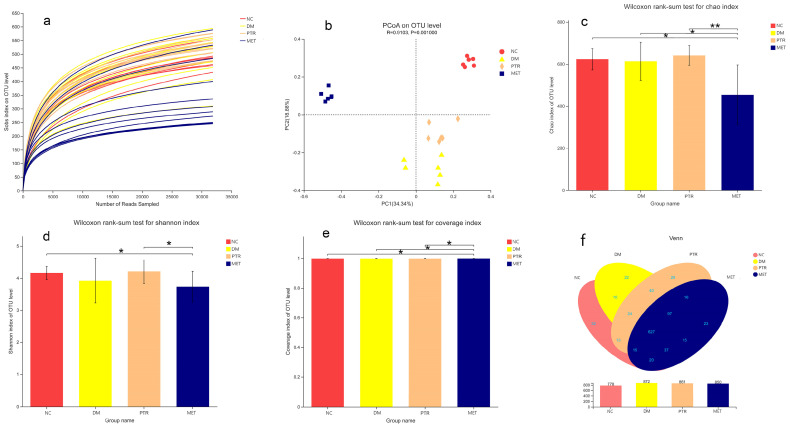
Effects on the overall structure of gut microbiota in db/db mice: (**a**) rarefaction curves among different groups; (**b**) Sobs index; (**c**) Chao index; (**d**) Shannon index; (**e**) unweighted unifrac-based principal coordinates analysis; (**f**) Venn diagram analysis at OUT level. Compared between two groups, the significance of the difference as described as * *p* < 0.05, ** *p* < 0.01.

A total of 2,866,188 intestinal content sequences were detected by 16S rRNA gene sequencing in this study. The dilution curve was close to the saturation plateau and the sequencing was reasonable (Figure 6a). Based on the 16S rRNA gene sequencing assessment, the Chao index reflects the richness of the gut microbiota, the Shannon index reflects community diversity, and the Coverage index refers to the coverage of the sample library. The higher the value, the higher the probability of detecting sequences in the sample. The indexes of each group of gut microbiota were analyzed as shown in Figure 6c–e. Compared with the NC group, there was no significant difference in the Chao index and Shannon index of the DM group, indicating that there was no significant change in the richness and diversity of the gut microbiota in db/db mice. Compared with the DM group, the Chao index and Shannon index of the PTR group were increased, but no significant difference was detected. Furthermore, a significant decrease in the Chao index and a moderate decrease in the Shannon index were identified in the MET group. Compared with the MET group, a significant increase in the Chao index, Shannon index, and Coverage index was identified in the PTR group, suggesting that the intake of PTR contributes to restoring the gut microbiota structure.

**Figure 7:**
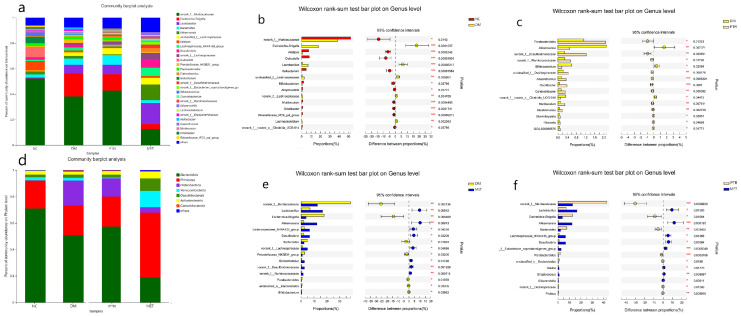
Effects on the overall composition of gut microbiota in db/db mice: (**a**) the relative abundance of gut microflora at the genus level; (**d**) the relative abundance of gut microflora at the phylum level; (**b**) Wilcoxon rank-sum test bar plot at the Genus level, the difference between the NC and DM group; (**c**) the difference between the DM and PTR groups; (**e**) the difference between the DM and MET groups; (**f**) the difference between the PTR and MET groups. Compared with the NC group or DM group, the significance of the difference was described as * *p* < 0.05, ** *p* < 0.01, *** *p* < 0.001.

In the Beta diversity analysis, the Bray–Curtis distance matrix PCoA analysis based on the OTU level showed a clear separation between the DM group and NC group. Furthermore, all groups were found to tend to be a distinct cluster, suggesting that each group in our study had a distinct gut microbiota composition at the OTU levels (Figure 6b). Based on OTU sequence analysis at a 97% similarity level, 778 OTU sequences were found in the NC group, 872 in the DM group, 861 in the PTR group, and 850 in the MET group (Figure 6f). At the phylum level, the dominant bacteria in the gut microbiota of the four groups of samples were Bacteroidota, Firmicutes, Proteobacteria, Desulfobacterota, Actinobacteriota, Verrucomicrobiota, and Campilobacterota (Figure 7d). Compared with the NC group, the relative abundance of *Firmicutes* in the DM group increased without a significant difference, while the relative abundance of *Bacteroidetes* decreased with a significant difference. However, there was no significant difference in the increase in the *Firmicutes/Bacteroidetes*(*F/B*) ratio. Compared with the DM group, the *F/B* ratio in the PTR group decreased but showed no significant difference, while the *F/B* ratio in the MET group increased and showed an extremely significant difference (*p* ≤ 0.001) (Table 2).

As shown in Figure 7a, the dominant bacteria in the gut microbiota of the four groups of samples at the genus level were *norank_f__Muribaculaceae*, *Escherichia-Shigella, Lactobacillus, unclassified_f__Lachnospiraceae, Akkermansia,* and *Alistipes*. The Wilcox rank-sum test was performed at the genus level to obtain the corresponding different bacterium. The differential bacterium between the NC group and DM group included *norank_f__Muribaculaceae, Escherichia-Shigella,Alistipes, Dubosiella,Lactobacillus,Ileibacterium,unclassified_f__Lachnospiraceae,Bifidobacterium, Alloprevotella,norank_f__Lachnospiraceae,Muribaculum,Odoribacter, Rikenellaceae_RC-9_gut_group, Lachnoclostridium,norank_f__norank_o__Clostridia_UCG-014*, *etc.* (Figure 7b). The differential bacterium between the PTR and DM groups included *Parabacteroides, Akkermansia, norank_f__Desulfovibrionaceae,norank_f__Ruminococcac-eae, Bifidobacterium,unclassified_f__Oscillospiraceae, Anaerotruncus, Oscillibacter, Colidextribacter,norank_f__norank_o__Clostridia_UCG-014,Muribaculum,Intestinimoas, Marvinbryantia,Rikenellagca-900066575*, etc. (Figure 7c). The differential bacterium between the MET group and DM group included *norank_f__Muribaculaceae,Lactobacillus,Escherichia-Shigella, Akkermansia,Lachnospira-ceae_NK4A136_group,Desulfovibrio, Bacteroides,norank_f__Lachnospiraceae,Prevotell—aceae_NK3B31_group, Enterorhabdus,norank_f__Desulfovibrionaceae,norank_f__Rumi-nococcaceae, Parabacteroides,unclassified_o__Bacteroidales, Bifidobacterium*, etc. (Figure 7e). The differential bacterium between the MET group and PTR group included *norank_f__Muribaculaceae,Lactobacillus,Escherichia-Shigella, Akkermansia, Bacteroides,Lachnospiraceae_NK4A136_group, Desulfovibrio,norank_f__Eubacterium_c-oprostanoligenes_group, Parabacteroides, unclassified_o__Bacteroidales, Blautia, Streptococcus, Alloprevotella, norank_f__Oscillospiraceae, Proteus, muribaculum,* etc. (Figure 7f).

### 2.6. Correlation Analysis of Gut Microbiota and Differential Metabolites

**Figure 8:**
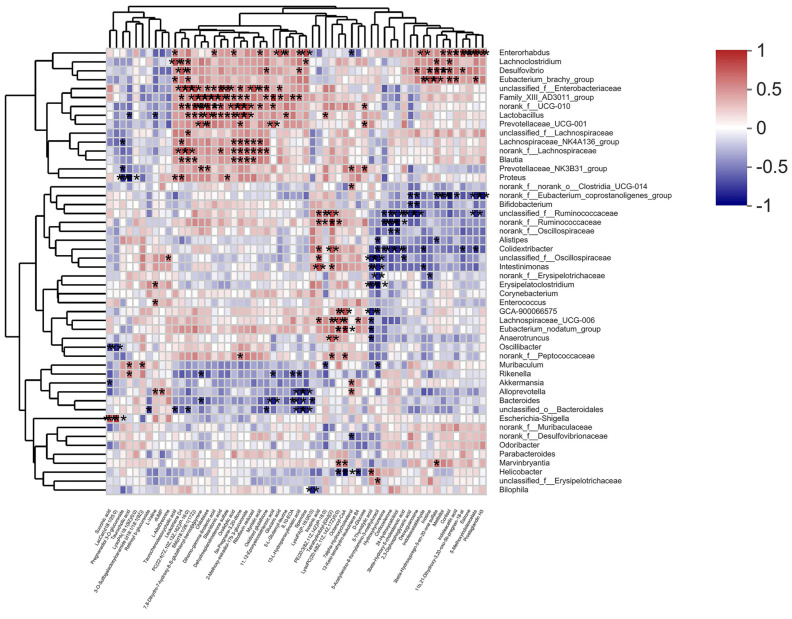
Spearman analysis between bacterial genera and microbial metabolites. The R values are represented by gradient colors, where pink and blue cells indicate positive and negative correlations, respectively. * *p* < 0.05, ** *p* < 0.01 and *** *p* < 0.001.

Spearman correlation analysis was used to calculate the correlation between the gut microbiota in the intestinal contents and the differential metabolites in the serum collected from db/db mice after being treated with PTR (Figure 8). Among them, *Muribaculum* was positively correlated with 3-O-Sulfogalactosylceramide (d18: 1/18: 1 (9Z)) and Taurocholic acid, while negatively correlated with 5-Acetylamino-6-formylamino-3-methyluracil and PE (20: −3 (8Z, 11Z, 14Z)/P-18: 0) (|r| > 0.5, *p* < 0.05, 0.01, 0.001). Additionally, *Desulfovibrio* was positively correlated with 3beta-Hydroxypregn-5-en-20-one sulfate, 11b, 21-Dihydroxy-3, 20-oxo-5b-pregnan-18-al, 13-L-Hydroperoxylinoleic acid, Leukotriene D4, Deoxyguanosine, Oxidized glutathione, Melibiitol, Cortisol, Inosine, and PC -(22:4(7Z,10Z,13Z,16Z)/P-16:0) (|r| > 0.5, *p* < 0.05, 0.01, 0.001). Lastly, *Blautia* was positively correlated with 5a-Pregnane-3, 20-dione, Riboflavin reduced, 2-Methoxy-estradiol-17b -3-glucuronide, Leukotriene D4, SM(d18:1/26:1(17Z)), Orotidylic acid, and PC -(22:4(7Z,10Z,13Z,16Z)/P-16:0) (|r| > 0.5, *p* < 0.05, 0.01, 0.001). The above results indicated that the changes in serum metabolites were closely linked to the changes in the gut microbiota after PTR supplementation.

## 3. Discussion

Since ancient times, Pueraria thomsonii Radix has been known as “Asian Ginseng”, and it is a traditional Chinese herbal medicine that can be used to raise body fluids and quench thirst according to the Chinese Pharmacopoeia [5]. Regarding physiological and biochemical indexes, the body weight and FBG of the DM group were significantly higher than that of the NC group in the first weekend of this study, indicating that db/db mice have met the conditions to serve as T2DM model mice after 3 days of adaptive feeding (Figure 2a,b). The typical symptoms of T2DM are polydipsia, polyuria, hyperglycemia, and weight loss [5]. Our results showed that the weight gain in the DM group is slower in the early stage and even leads to weight loss in the later stage, while the FBG increases faster, which is consistent with the symptom description of “three more and one less” in T2DM reported by multiple scholars in the prophase [14,15]. After the treatment with PTR and metformin, there was no significant difference in the body weight of each group of db/db mice at the weekend of every week. In addition, FBG saw a significant decrease from the third weekend of treatment. Moreover, physiological and biochemical indexes such as FINS, HOMA-IR, UGLU, and UACR in the PTR and MET groups were significantly lower than those in the DM group at the end of the ninth week after euthanasia, as well as pancreatic tissue damage, which indicated that PTR and metformin improve insulin resistance and leptin resistance in db/db mice. This is in line with previous studies showing that the thirst-quenching effects of PTR are associated with improved insulin resistance [15]. Metformin is often used to treat T2DM patients who are unsatisfied with dietary control alone and is also commonly used in the development of new anti-diabetic drugs to evaluate the efficacy of new drugs against diabetes. In experimental diabetes models, metformin was effective in downregulating blood glucose levels and improving insulin resistance in diabetic mice and rats. In this study, our pharmacodynamic experiments showed that PTR is similar to metformin in treating T2DM by decreasing FBG, FINS, HOMA-IR, UGLU, UACR, and pancreatic tissue damage in db/db mice. This further suggests that the anti-diabetic potential of PTR is in urgent need of further clinical studies, or the use of dietary therapy to control diabetes.

Regulating metabolism is an effective measure for the treatment of diabetes. In this study, PTR reversed 74 of the 109 differential metabolites in db/db mice, mainly including purines, pyrimidines, glycerophospholipids, and steroids. Purine and pyrimidine metabolism are central to the adenosine system and play a key role in the regulation of glucose homeostasis as well as the pathophysiology of diabetes [16,17]. In this study, PTR affected the purine metabolic pathway by regulating the levels of Inosine, Inosinic acid, and Deoxyguanosine in db/db mice, and the pyrimidine metabolic pathway by regulating the concentrations of Orotidylic acid, dUMP, and 5-Thymidylic acid, thereby improving T2DM. Abnormal glycerophospholipid metabolism is a clinical feature of adult-onset T2DM [18]. In this study, PTR could modulate PE (20:3(8Z,11Z,14Z)/P-18:0), PC (22:4(7Z,10Z,13Z,16Z)/P-16:0), and LysoPA (18:1(9Z)/0:0) concentrations in db/db mice. Previous studies have shown that the up-regulated release of cortisol is implicated in a hyperglycemic response and associated with diabetes-related distress in people with T2DM [19,20], and corticosterone may induce insulin resistance in rat hepatocytes [21]. In this experiment, db/db mice showed a significant increase in serum cortisol and a strong positive correlation with *Desulfovibrio*, a harmful bacteria in the intestinal tract whose increased abundance can cause inflammation and insulin resistance [22], while PTR could effectively reduce the levels of serum cortisol and *Desulfovibrio* in the intestinal tract, thus effectively alleviating T2DM.

There is growing evidence that dysbiosis of the intestinal flora is a typical feature of the pathogenesis of diabetes. Clinical studies have found that *Bifidobacterium, Bacillus* spp., *Clostridium pretense*, and *Ackermannia* spp. are negatively associated with T2DM, and that *Clostridium tumefaciens, Clostridium perfringens,* and *Braunschweiger* spp. are positively associated with T2DM. In animal models of experimental diabetes, the species and number of beneficial bacteria were significantly lower, while pathogenic bacteria were significantly higher [23]. A study has demonstrated that the *Plukenetia volubilis* L. leaf significantly increased *Muribaculum* abundance in db/db mice, a beneficial bacterium that can improve inflammation, dyslipidemia, and glucose intolerance [24,25]. In this study, we found that PTR significantly upregulated the abundance of *Muribaculum*, *Norank_f_Muribaculaceae*, and *Parabacteroides* in db/db mice, while significantly reducing the abundance of the harmful bacterium *Desulfovibrio*. *Norank_f_Muribaculaceae* is a beneficial dominant bacterium in the mouse intestine and is negatively correlated with the glucose levels of postprandial blood [26]. *Parabacteroides* is a beneficial bacterium that significantly improves insulin resistance and has been shown in vitro to activate the intestinal gluconeogenic pathway through the conversion of succinate, thereby exerting a hypoglycaemic effect [27]. Previous studies have reported a positive association between the *F/B* ratio and the incidence of obesity and diabetes [24,25]. Some studies have shown that obese animals and humans exhibit higher *F/B* ratios in their gut microbiota compared to individuals with normal body mass [28]. In our study, compared with the NC group, the relative abundance of *Firmicutes* in the DM group increased without a significant difference, while the relative abundance of *Bacteroidetes* decreased with a significant difference. However, there was no significant difference in the decrease in the F/B ratio. However, the F/B ratio in the PTR group decreased with no significant difference compared with DM group, but the decreasing trend of the F/B ratio after PTR administration was consistent with other anti-T2DM drugs previously reported.

**Figure 9:**
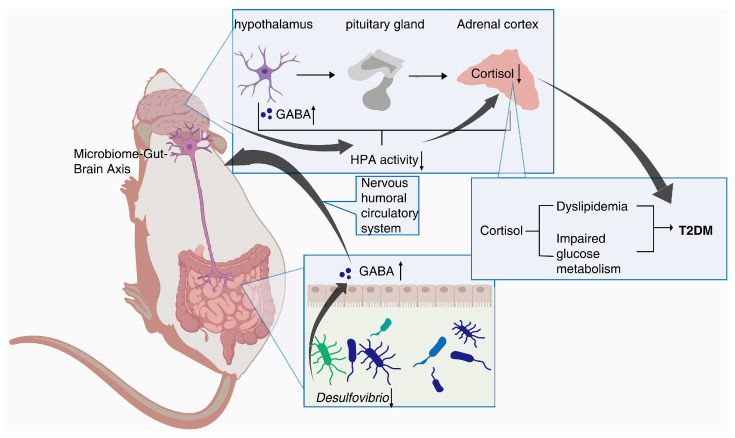
The schematic of mechanism of action of PTR.

In addition, Spearman’s correlation analysis further revealed a positive correlation between *Desulfovibrio* and Cortisol in db/db mice. Cortisol is an important product of the steroid hormone biosynthetic pathway and is considered an active indicator of the hypothalamic–pituitary–adrenal (HPA) axis. A recent study has shown that cortisol is associated with dyslipidemia and impaired glucose metabolism [29]. *Desulfovibrio* has been reported to be negatively correlated with γ-aminobutyric acid (GABA) [30], which can inhibit glucagon secretion and enhance insulin secretion [31]. Notably, hypothalamic Cortisol concentrations are influenced by GABA, and reduced GABA levels lead to over-activation of the HPA axis, resulting in increased Cortisol levels [32,33]. Recent research hotspots have reported a stronger bidirectional communication between the gut and brain through the nervous, endocrine, and immune systems, i.e., MGB [34,35]. In this experiment, PTR reduced the abundance of *Desulfovibrio* and the levels of Cortisol metabolism. Therefore, we speculate that PTR administration resulted in a decrease in *Desulfovibrio* abundance, leading to an increase in GABA levels in the gut, which in turn inhibited HPA axis activation via the enteric nervous system (ENS) via the MGB pathway, contributing to a decrease in Cortisol metabolism levels (Figure 9). This is the focus of our subsequent studies, and perhaps related studies will be reported in the near future.

Regarding the chemical composition, the content of polysaccharides and puerarin were the highest two components, and were significantly higher than the sum of other polyphenols in this study (Table 1), which is consistent with previous research that found that both polysaccharides and polyphenols improve T2DM [7,8,9,10,11]. In addition, our collaborative research group found that polysaccharides from Puerariae thomsonii Radix can improve fatty liver and hyperglycemia by regulating the intestinal flora and metabolites [36,37]. In this study, we found that PTR can reduce the *F/B* ratio and regulate three beneficial bacteria and one harmful bacterium to exert hypoglycemic effects through the regulation of the nervous system, possibly mainly due to the role of its polysaccharides. However, more previous research supports that polyphenols can exert hypoglycemic effects by improving insulin resistance [7,8,9,10,11]. Therefore, we hypothesized that PTR, as a water extract of *Pueraria thomsonii* Radix, contains both polyphenols and polysaccharides, which may play a synergistic role in improving T2DM. Next, we will collaborate with another research group to investigate the differences in drug efficacy between the polysaccharide group, polyphenol group, and PTR group (containing both polysaccharides and polyphenols), in order to elucidate whether polyphenols and polysaccharides have synergistic effects and differences in improving T2DM.

## 4. Materials and Methods

### 4.1. Preparation of PTR

*Pueraria thomsonii* Radix, purchased from Jiangzhong Traditional Chinese Medicine Co., Ltd. (Nanchang, China), was identified as the dried root of the legume plant *Pueraria Thomsonii* Benth by Professor Fei Ge and Professor Ronghua Liu of Jiangxi University of Chinese Medicine. PTR was extracted by Jiangxi Xinglin Baima Pharmaceutical Co., Ltd., Nanchang, China. A total of 200 kg of raw material was extracted 3 times continuously with 15 times the amount of water each time, filtered in a basket, combined with the filtrate, and concentrated under reduced pressure. After vacuum drying, 29.4 kg of PTR (yield: 14.70%) was obtained by crushing, which was used for subsequent animal experiments and preparation of the test solution. In our previous experiments, the UPLC-Q-TOF/MS technique was used to analyze the absorbed components of PTR in rat blood, mainly including various polyphenols (Supplementary Table S1) [38]. In this experiment, nine kinds of polyphenols and total polysaccharides related to T2DM were selected for qualitative analysis through previous literature investigation [7,8,9,10,11].

### 4.2. Preparation of Test Solution

PTR was converted into 2 g of raw material of *Pueraria Thomsonii* Radix. Afterwards, it was placed in a conical flask with a stopper, added 50% methanol (50 mL), weighed, heated in an ultrasonic bath for 60 min, cooled, weighed again, made up the lost weight with 50% methanol, shook well, and filtered through a PTFE membrane (0.22 μm) (Tianjin Zinteng Experimental Equipment Co., Ltd., Tianjin, China). The filtered solution of PTR was tested for the presence of various polyphenols (Supplementary Table S1) [38].

Water extraction and the alcohol precipitation method were used for the extraction and separation of polysaccharides from PTR. PTR was carefully weighed (0.0441 g), anhydrous ethanol was slowly added to the sample until no more precipitate was produced, and the samples were shaken and mixed. After 30 min of ultrasonic extraction, the samples were centrifuged for 10 min, the supernatant was taken again, and the insoluble substance was mixed twice. The insoluble substance was washed with 10 mL of 80% ethanol solution and centrifuged for 10 min. We transferred the insoluble substance to a beaker, added 50 mL of ultra-pure water to dissolve, sonicated for 30 min, and repeated twice. We cooled, filtered, transferred the filtrate in a 100 mL volumetric flask, washed the residue 2~3 times, and held the volume of ultra-pure water to obtain the solution to be tested for the determination of polysaccharides.

### 4.3. Preparation of Reference Solution

The nine polyphenols were purchased from Shanghai Meryer Chemical Technology Co., Ltd., Shanghai, China. We accurately weighed the above substances, added methanol to prepare a mixed reference stock solution with corresponding mass concentration, and diluted it step by step to obtain a series of mixed reference solutions with nine polyphenols in different mass concentrations.

D-anhydrous glucose standard (Shanghai Meryer Chemical Technology Co., Ltd., Shanghai, China) was taken, accurately weighed, dissolved in distilled water to volume, and prepared into a D-anhydrous glucose reference solution with a concentration of 1 mg/mL for the determination of polysaccharide content. The standard d-anhydrous glucose was weighed accurately, dissolved in distilled water to scale, and prepared into a 1 mg/mL D-glucose control solution, which was used to draw the standard curve and determine the content of polysaccharides.

### 4.4. Determination of PRT Active Ingredient Content

The quantitative analysis of polyphenols was performed on an Agilent 1260 Infinity II liquid chromatography system using a Cosmosil cholester (Santa Clara, CA, USA, 250 mm × 4.6 mm, 5 μm). The chromatographic column was used for HPLC analysis with a flow rate of 1 mL/min. The injection volume was 10 µL. The column temperature was 30 °C. The DAD detection wavelength was 254 nm. The mobile phase used was 0.1% formic acid in water (A) and acetonitrile (B). The gradient elution was as follows: 0–3 min, 5–11% (B); 3–15 min, 11% (B); 15–25 min, 11–15% (B); 25–35 min, 15–20% (B); 35–40 min, 20–30% (B); 40–55 min, 30–50% (B); and 55–65 min, 50–80% (B). Precision measurement: the mixed reference solution of a certain concentration prepared in Section 4.3 was accurately injected and repeated six times. Repeatability test: a total of six injections of the test solution were prepared for the same sample. Stability test: a sample was taken and injected at 0, 1, 2, 4, 16, and 24 h. A calibration curve for linearity analyses was established by injecting each concentration of the control solution (reference solutions in Section 4.3) under the chromatographic conditions indicated above, whereby the peak area (Y) corresponded to the ordinate and the control injection amount (X) indicated the abscissa. The peak areas of nine polyphenols were substituted into linear equations to calculate the percentage contents of each of the nine polyphenols of PTR.

The content of the polysaccharides in PTR was determined by colorimetry using a microplate detection system with SpectraMax 190 microplate (Shanghai Meigu Molecular Instrument Co., Ltd., Shanghai, China). The specific approach was as follows: firstly, precision, repeatability, and stability experiments were conducted. Subsequently, we diluted the D-glucose reference solution in Section 4.3 to six gradients of the concentrated solution, added 200 μL of 5% phenol solution to each 200 μL, then quickly added 1 mL of concentrated sulfuric acid, let it stand for 10 min, mixed well with vortex oscillation, used 1.0 mL of ultrapure water as the blank control, and then placed the test tube in a water bath at 30 °C for 20 min. We measured the absorbance at 490 nm, using the concentration (mg/mL) as the horizontal axis and the absorbance value as the vertical axis to draw a standard curve. Under the same conditions, we accurately absorbed 200 μL of the PTR test solution prepared in Section 4.2, used distilled water as a blank control, and measured the absorbance of the PTR test solution using the same method as the previous standard curve. Finally, we converted the total polysaccharides content using the correction coefficient of the glucose standard.

### 4.5. Animals and Sample Collection

The treatment of animals during the experiment was in accordance with the eighth edition of the Regulations on the Management of Laboratory Animals of China and was approved by the Ethics Committee of the Laboratory Animal Science and Technology Center of Jiangxi University of Chinese Medicine (Approval No. JZLLSC20210075). The 8-week-old male db/db mice and db/m mice (raised under the same conditions, age, and homology in an SPF environment) were obtained from Changzhou Cavins Biotech Corporation, Ltd. [Animal Certificate Number: SCXK (Su) 2016-0013]. Measuring and comparing FBG of db/dm and db/m mice, we confirmed that db/m mice could serve as T2DM model mice and subsequently grouped the animals as follows: 11 db/m mice were used as Normal Control (NC) group and 39 db/db mice were modeled for T2DM and randomly divided into three groups, with 13 mice in each: Diabetes Control (DM) group, PTR group, and Metformin (MET) group. Then, they were raised in the protective environment of the Animal Science and Technology Experimental Center of Jiangxi University of Chinese Medicine (humidity: 50 ± 5%, temperature: 22 ± 2 °C, and light-dark cycle for 12 h).

After three days of adaptive feeding, the body weight and FBG of each group were measured as their first weekend data, then each group was given corresponding drugs by intragastric administration according to their weight, once a day for 8 weeks, and their weight and FBG were measured at the end of every week. Namely, the mice in the NC group and DM group were intragastrically administered with distilled water, the mice in the PTR group were intragastrically administered with PTR 0.89 g/kg/d (according to the formula of human and mouse body surface area, the most commonly used dosage for human administration and the yield of extract powder prepared from raw drug), and the mice in the MET group were intragastrically administered with the original drug of metformin 0.364 g/kg/d (Sino American Shanghai Squibb Pharmaceutical Co., Ltd., China).

On the night before the ninth weekend, we started fasting and water prohibition at 18:00. On the morning of the ninth weekend, about 50 μL of blood was collected from the orbital venous plexus of the mice and put into common EP tubes for the determination of FINS. One hour after the last administration, mice were anesthetized and whole blood was collected from abdominal aorta into common EP tubes and centrifuged at 8000 r/min for 15 min in a cryogenic centrifuge at 4 °C. After centrifugation, the supernatant was collected and stored at −80 °C for serum metabolomics study. After euthanizing, samples of the pancreatic and intestinal contents were carefully collected and then flash-frozen in liquid nitrogen and stored at −80 °C for subsequent experimental analysis.

### 4.6. Analysis of Physiological and Biochemical Indexes

The weekly body weight of mice was measured using an electronic analytical balance (Beijing Sartorius Scientific Instruments Co., Ltd., Beijing, China). The levels of FBG were measured weekly using a blood glucose meter and a blood glucose strip (Roche Blood Glucose Health Care). FINS were measured using the Mouse Insulin (INS) Elisa kit (Nanjing Jiancheng Bioengineering Institute, China). HOMA-IR was calculated using the following formula: FBG × FINS/22.5. UGLU was measured using an automatic biochemical analyzer (Tokyo, Japan, Hitachi7180). UACR was measured by an automatic biochemical analyzer from Catalyst (Wellington, New Zealand).

### 4.7. Observation of Pancreatic Hematoxylin-Eosin (HE) Staining

The pancreatic samples were fixed in a 10% formalin solution. A paraffin-embedding machine and microtome (Leica, Wetzlar, Germany) were used to embed and slice the tissue to a thickness of 4 μm. The sections were routinely stained with HE, sealed with neutral gum, and mounted on a glass slide and examined under a light microscope (Mshot, Beijing, China) with 400× total magnification. Based on the pathological changes in the understudied tissues, the histological sections were rated approximately on a scale from 0 to 4, with no change (0), minimal changes (+1), mild changes (+2), moderate changes (+3), and severe changes (+4).

### 4.8. Untargeted Metabolomics Analysis

We thawed the frozen serum samples naturally, took 50 μL of serum, added 200 μL of the methanol working solution (containing 10.06 µg/mL 2-chloro-L-phenylalanine), mixed them using the MTV-100 vortex mixer (Hangzhou Aosheng Group Co., Ltd., Hangzhou, China) for 30 s, and after standing for 10 min, centrifuged at 12,000 r/min for 15 min at 4 °C. The supernatant was taken for untargeted metabolomic analysis in UPLC-IM-Q-TOF-MS (UPLC I-CLASS liquid chromatography system and SYNAPT G2-Si mass spectrometer, Waters, MA, USA). In addition, quality control (QC) samples were prepared by taking an equal amount from each frozen serum sample, vortexed and mixed according to the above method.

Liquid chromatographic separation of the samples was achieved using an ACQUITY UPLC BEN C18 column (2.1ITY UPLC BEm, Waters Corporation). The injection volume was 2 µL, the column temperature was 40 °C, the flow rate was 0.35 mL/min, the eluent A was water containing 0.1% formic acid, and the eluent B was acetonitrile. Solvent gradient setting in positive ion mode: 0–0.2 min, 5% B; 0.2–2 min, 5–20% B; 2–7 min, 20–50% B; 7–17 min, 50–65% B; 17–22 min, 65–80% B; 22–23 min, 80–95% B; 23–26 min, 95–5% B; negative ion mode: 0–0.2 min, 5% B; 0.2–3 min, 5–20% B; 3–5 min, 20–45% B; 5–7 min, 45–55% B; 7–13 min, 55–65% B; 13–16 min, 65% B; 16–21 min, 65–80% B; 21–23 min, 80–95% B; 23–26 min, 95–5% B.

An ESI ion source was used to carry out data acquisition in positive and negative ion mode in HDMSE mode. The mass range (*m*/*z*) was 50–1200 Da, ion source temperature was 120 °C, curtain rate cone gas flow rate was 50 L/h, desolvation gas flow rate was 800 L/h, desolvation temperature was 400 °C, cone voltage was 40 V, collision energy was 20–40 V, scan time and inter-scan delay were 0.3 s and 0.015 s, capillary ESI+ was 3 kV, and ESI- was 2.3 kV. In order to ensure the accuracy and reproducibility of the experimental data, the standard product sodium formate was used to establish the mass axis standard curve, and at the same time, leucine enkephalin was used for real-time mass correction, and polyalanine (purchased from Sigma p/n P9003) was used to perform CCS Correction.

In this experiment, in order to ensure the stability of the analysis system as a whole, method validation was performed using quality control (QC) samples. Before collecting each group of samples, QC samples were advanced five times to balance the instrument. After stabilization, one QC sample was injected for every five experimental samples to monitor the operation of the system in real-time. The real-time monitoring system should be run to analyze the data in real-time and evaluate the data quality. Finally, the peak intensities of 10 typical mass spectrometry peaks were analyzed, the value of relative standard deviation (RSD) was used to confirm that the instrument injection was stable, and the method was reproducible.

The mass spectrometry raw data obtained by UPLC-IM-QTOF-MS were imported into Progenesis QI V2.0 software (QI, Waters, USA) for normalization processing, and then the data matrix was exported and saved. After grouping the data, we exported the data to the EZinfo3.0 (Waters, USA) software to establish labels (VIP > 1 and *p* < 0.05) to screen differential metabolites. The SIMCA14.1 (Swedish Umetrics Company) software was used to perform principal component analysis (PCA) and orthogonal partial least squares discriminant analysis (OPLS-DA) on the data obtained in the previous step. According to the data of the mass-to-charge ratio, retention time, CCS value, and secondary mass spectrometry fragments of the differential metabolites, they were matched and identified in the HMDB database, and then were analyzed by Pathway Analysis in the Metabo Analyst 5.0 (https://www.metaboanalyst.ca) (accessed on 18 October 2022) online database. The identified differential metabolites were enriched for metabolic pathways, and the differential metabolites were finally determined to obtain metabolic pathways and conduct pathway analysis.

### 4.9. Gut Microbiota Analysis

Gut microbiota analysis was performed by Shanghai Magi Biomedical Technology Co., LTD. (Shanghai, China). We thawed the frozen samples of the intestinal contents naturally and DNA was extracted from differential samples of intestinal contents using E.Z.N.A.^®^ Soil DNA Kit (Omega Biotek, Norcross, GA, USA). The quality of DNA extraction was examined using 1% agarose gel electrophoresis, and the concentration and purity of the DNA were determined using a NanoDrop2000 ultramicro spectrophotometer (Thermo Scientific, Wilmington, USA). The hypervariable region V3-V4 of the bacterial 16S rRNA gene was amplified with primer pairs 338F (5’-ACTCCTACGGGAGGCAGCAG-3’) and 806R (5’-GGACTACHVGGGTWTCTAAT-3’) by an ABI GeneAmp^®^ 9700 PCR thermocycler (ABI, California, USA). There were three replicates for each sample. The PCR products of each sample were mixed in triplicate and then the PCR products were recovered on 2% agarose gel. AxyPrep DNA Gel Extraction Kit (Axygen Biosciences, Union City, CA, USA) was used to purify the recovered product and detect it by 2% agarose gel electrophoresis. Quantus™ Fluorometer (Promega, Wisconsin, USA) was used to detect and quantify the recovered products. The NEXTflexTM Rapid DNA-Seq Kit (Bioo Scientific, Texas, USA) was used for library construction. Sequencing was performed using Illumina’s Miseq PE300 platform. The fastp software (https://github.com/OpenGene/fastp, version 0.20.0) (accessed on 5 November 2022) was used for quality control of original sequencing sequence. FLASH software (http://www.cbcb.umd.edu/software/flash, version 1.2.7) (accessed on 5 November 2022) was used to pick up the fight. UPARSE software (http://drive5.com/uparse/,Version 7.1) (accessed on 5 November 2022) was used to cluster OTU sequences according to a 97% similarity. RDP classifier (http://rdp.cme.msu.edu/, version 2.2) (accessed on 5 November 2022) was used to annotate each sequence for species classification. The comparison threshold was set to 70% for Silva 16S rRNA database (V138).

Statistical analyses. These values were defined as mean ± standard deviation (SD). The difference between the two groups of metabolites was calculated using student’s *t*-test by GraphPad prism 9 (GraphPad Software Inc., La Jolla, CA, USA). The difference between the two groups of gut microbiota was calculated using the Wilcox rank-sum test. Compared with the NC group or DM group, the significance of the difference was described as * *p* < 0.05, ** *p* < 0.01, and *** *p* < 0.001.

## Figures and Tables

**Table 1:** The contents of nine polyphenols and total polysaccharides of PTR.

	Sample	PTR (µg/g)
Isoflavones		
3′-hydroxy puerarin		44.27 ± 1.03
puerarin		3339.05 ± 8.56
puerarin apioside		323.91 ± 2.14
daidzin		955.92 ± 5.68
genistin		53.42 ± 1.32
Ononin		1.94 ± 0.09
daidzein		351.20 ± 1.15
genistein		8.82 ± 0.41
formononetin		4.63 ± 0.29
total polysaccharides		2050.36 ± 60.22

**Table 2:** Relative abundance values of several important bacteria at the phylum level.

Sample	NC	DM	PTR	MET
*Bacteroidota*	70.91 ± 13.58 **	50.62 ± 17.00	57.21 ± 11.81	18.76 ± 24.65 **
*Firmicutes*	21.14 ± 9.919	22.66 ± 12.30	22.82 ± 9.377	49.09 ± 18.98 **
*F/B*	0.2896 ± 0.1440	0.5246 ± 0.4121	0.4259 ± 0.2092	25.40 ± 5.779 ***

n = 11; ** *p* < 0.01 and *** *p* < 0.001. Compared with the DM group.

## Data Availability

The data used to support the findings of this study are available from the corresponding author upon request.

## Supplementary Materials

The following supporting information can be downloaded at: https://www.mdpi.com/article/10.3390/molecules28227471/s1, Supplementary Table S1: LC-MS data of the absorbed components of PTR in rat blood; Supplementary Table S2: Differential metabolites; Supplementary Table S3: Differential metabolites for PTR callbacks; Supplementary Table S4: Differential metabolites for Metformin callbacks; Supplementary Table S5: Analysis of PTR metabolic pathway enrichment; Supplementary Table S6: Analysis of Metformin metabolic pathway enrichment.

## Abbreviations

PTR	*Pueraria thomsonii* Radix water extract
T2DM	Type 2 diabetes mellitus
FBG	Fasting blood glucose
UGLU	Urinary glucose
HOMA-IR	Homeostasis model assessment insulin resistance
UACR	Urinary albumin/creatinine ratio
CCS	Collision cross section
OPLS-DA	Orthogonal partial least squares discriminate analysis
GABA	γ-aminobutyric acid
HPA	Hypothalamic-pituitary-adrenal
MGB	The microbiota-gut-brain axis
*F/B*	*Firmicutes/Bacteroidetes*
HE	Hematoxylin-eosin
QC	Quality control
RSD	Relative standard deviation
