# Supplementary material for: Pueraria thomsonii Radix Water Extract Alleviate Type 2 Diabetes Mellitus in db/db Mice through Comprehensive Regulation of Metabolism and Gut Microbiota
Source: Molecules. 2023 Nov 7;28(22):7471. doi: 10.3390/molecules28227471 (PMC10673130; doi:10.3390/molecules28227471)
Supplement: Supplementary file 1 [file molecules-28-07471-s001.zip › Supplementary Table S1.pdf]

**Supplementary Table S1**.LC-MS data of the absorbed components of PTR in rat blood[12][#]

| modle | NO | Retention time | Name | Formula | Experimental Mass | Secondary fragment ions | Mass Error |
|---|---|---|---|---|---|---|---|
| [M-H]- | F1 | 9.38 | puerarin-4'-O-β-D-glucopyranoside* | C27H30O14 | 577.1571 | 457.1132 | 1.4 |
| [M-H]- | F2 | 14.51 | puerarin* | C21H20O9 | 415.1039 | 295.0602,277.0489,267.0644 | -2.5 |
| [M-H]- | F3 | 15.19 | puerarin-6´´-O-xyloside* | C26H28O13 | 547.1444 | 295.0570 | -2.5 |
| [M-H]- | F4 | 15.72 | 3´-methoxy puerarin* | C22H22O10 | 445.1123 | 325.0699,310.0476,297.0738,282.0521 | -3.8 |
| [M-H]- | F5 | 15.91 | puerarin apioside* | C26H28O13 | 547.1455 | 295.0588,267.0642 | -0.5 |
| [M-H]- | F6 | 17.65 | p-Hydroxycinnamic acid* | C9H8O3 | 163.0395 | 119.0495 | -3.6 |
| [M+H]+ | F7 | 17.82 | daidzin* | C21H20O9 | 417.1172 | 255.0646 | -2.0 |
| [M-H]- | F8 | 23.91 | salicylic acid* | C7H6O3 | 137.0244 | 93.0348 | -0.1 |
| [M+H]+ | F9 | 29.53 | ononin* | C22H22O9 | 431.1334 | 269.0797 | -0.5 |
| [M-H]- | F10 | 30.74 | daidzein* | C15H10O4 | 253.0495 | 224.0459,208.0507 | -4.4 |
| [M-H]- | F11 | 37.61 | naringenin* | C15H12O5 | 271.0596 | 151.0011,119.0504 | -5.9 |
| [M-H]- | F12 | 37.88 | genistein* | C15H10O5 | 269.0454 | 269.0447 | -0.6 |
| [M-H]- | F13 | 39.19 | isoliquiritigenin* | C15H12O4 | 255.0650 | 135.0078,119.0510,91.0196 | -4.9 |
| [M-H]- | F14 | 39.45 | formononetin* | C16H12O4 | 267.0648 | 252.0410,223.1684,195.0436 | -5.7 |
| [M-H]- | F15 | 40.38 | biochanin A* | C16H12O5 | 283.0600 | 267.0286,240.0426,239.0321,211.0366 | -4.3 |

Note *: indicates confirmation by comparison with reference material; [12][#]: Adapted from Honghua Yu, Xiaowei Meng, and **Jiarong Li**(2022 China journal of Chinese materia medica).
