# Supplementary material for: Pueraria thomsonii Radix Water Extract Alleviate Type 2 Diabetes Mellitus in db/db Mice through Comprehensive Regulation of Metabolism and Gut Microbiota
Source: Molecules. 2023 Nov 7;28(22):7471. doi: 10.3390/molecules28227471 (PMC10673130; doi:10.3390/molecules28227471)
Supplement: Supplementary file 1 [file molecules-28-07471-s001.zip › Supplementary Table S2.pdf]

Supplementary Table S2. Differential metabolites

modle	NO	HMDB ID	Retention time	Name	Formula	Experimental Mass	Ion Mode	Mass Error	MS/MS quantity	levels
ESI-	P1	0003871	6.72	13-L-Hydroperoxylinoleic acid	C18H32O4	333.205145	M+Na-2H	1.33761043	12	↑
	P2	0003871	6.23	13-L-Hydroperoxylinoleic acid	C18H32O4	333.2045847	M+Na-2H	-0.456798865	14	↑
	P3	0004701	9.41	9,10-EOA	C18H32O3	295.2277627	M-H	-0.35696978	8	↑
	P4	0004702	9.41	12,13-EpOME	C18H32O3	295.2277627	M-H	-0.35696978	8	↑
	P5	0001256	8.42	Spermine	C10H26N4	239.164631	M+K-2H	1.379597855	2	↑
	P6	0005050	6.90	15H-11,12-EETA	C20H32O4	371.2025	M+Cl	-0.060850098	18	↓
	P7	0004673	18.15	11,12-Epoxyeicosatrienoic acid	C20H32O3	365.2282	M+Na	3.620233869	6	↑
	P8	0011134	18.15	5-HETE	C20H32O3	365.2282	M+Na	3.620233869	6	↑
	P9	0003080	20.00	Leukotriene D4	C25H40N2O6S	533.2077227	M+K-2H	-3.204984531	3	↑
	P10	0013040	6.12	Prostaglandin H3	C20H30O5	331.1900557	M-H2O-H	-4.0754055	19	↑
	P11	0004673	5.82	11,12-Epoxyeicosatrienoic acid	C20H32O3	365.2318088	M-H	-4.80572325	16	↑
	P12	0011136	11.06	19(S)-HETE	C20H32O3	319.2269203	M-H	-2.960685962	3	↓
	P13	0013461	19.75	SM(d18:1/26:1(17Z))	C49H97N2O6P	877.6558603	M+K-2H	-1.392091422	2	↑
	P14	0003759	18.60	5a-Pregnane-3,20-dione	C21H32O2	677.4522447	M-H	-0.312038182	4	↑
	P15	0060382	5.40	3beta-Hydroxypregn-5-en-20-one sulfate	C21H32O5S	415.1942706	M+F	-1.401950917	5	↑
	P16	0006765	19.99	2-Methoxy-estradiol-17b 3-glucuronide	C25H34O9	459.2041349	M-H2O-H	3.540906446	3	↑
	P17	0001032	20.74	Dehydroepiandrosterone sulfate	C19H28O5S	367.1572338	M-H	-3.353725684	4	↑
	P18	0000063	5.58	Cortisol	C21H30O5	361.200367	M-H	-4.63983385	9	↑
	P19	0006754	5.96	11b,21-Dihydroxy-3,20-oxo-5b-pregnan-18-al	C21H32O5	363.2161356	M-H	-4.28880625	14	↑
	P20	0000145	5.94	Estrone	C18H22O2	329.1792614	M-H	0.88782721	7	↑

P21	0000063	5.58	Cortisol	C21H30O5	429.1874587	M-H	-4.92991872	9	↑
P22	0000063	14.33	Cortisol	C21H30O5	361.1990629	M-H	-4.63983385	5	↓
P23	0006754	9.91	11b,21-Dihydroxy-3,20-oxo-5b-pregnan-18-al	C21H32O5	385.1982038	M+Na-2H	-3.948506422	7	↑
P24	0004195	4.51	5-L-Glutamyl-taurine	C7H14N2O6S	253.0511982	M-H	2.719435723	10	↓
P25	0000036	5.61	Taurocholic acid	C26H45NO7S	514.283118	M-H	-3.485294884	7	↑
P26	0004195	5.11	5-L-Glutamyl-taurine	C7H14N2O6S	253.0507985	M-H	3.218921065	10	↓
P27	0004096	4.47	5-Methoxyindoleacetate	C11H11NO3	204.0659817	M-H	-3.096997856	12	↑
P28	0010396	9.92	LysoPC(20:4(8Z,11Z,14Z,17Z)/0:0)	C28H50NO7P	578.3002386	M+Cl	-3.041022578	2	↑
P29	0001190	4.49	Indoleacetaldehyde	C10H9NO	158.0603531	M-H	-3.99269806	3	↑
P30	0060484	1.05	Indolepyruvate	C11H9NO3	238.0232464	M+Cl	-1.564934306	2	↓
P31	0003337	16.56	Oxidized glutathione	C20H32N6O12S2	611.1341823	M-H	-0.249004006	2	↑
P32	0000806	18.62	Myristic acid	C14H28O2	249.1832989	M+Na-2H	-1.310307239	2	↑
P33	0000218	1.03	Orotidylic acid	C10H13N2O11P	402.9869625	M+Cl	4.172100199	6	↑
P34	0060423	18.44	7,8-Dihydro-7-hydroxy-8-S-glutathionyl-benzo[a]pyrene	C30H29N3O7S	610.1423377	M+Cl	0.547817024	12	↑
P35	0010384	17.03	LysoPC(18:0)	C26H54NO7P	522.3553686	M-H	-2.187111707	2	↓
P36	0003556	17.38	Chitobiose	C15H26N2O12	425.1417596	M-H	1.083517545	7	↑
P37	0006547	18.44	Stearidonic acid	C18H28O2	597.40825	M-H	0.449710047	3	↑
P38	0006733	22.64	CE(22:6(4Z,7Z,10Z,13Z,16Z,19Z))	C49H76O2	717.5681936	M-H	1.063394566	7	↓
P39	0001294	22.30	2,3-Diphosphoglyceric acid	C3H8O10P2	286.9382879	M+H	-3.307086773	13	↑
P40	0000085	22.29	Deoxyguanosine	C10H13N5O4	288.0703242	M+Na-2H	-4.108974939	8	↑
P41	0009116	23.08	PE(18:2(9Z,12Z)/P-18:1(11Z))	C41H76NO7P	746.5109327	M+Na-2H	0.447270627	4	↓
P42	0009117	23.08	PE(18:2(9Z,12Z)/P-18:1(9Z))	C41H76NO7P	746.5109327	M+Na-2H	0.447270627	4	↓
P43	0008198	24.28	PC(18:3(9Z,12Z,15Z)/15:0)	C41H76NO8P	722.5097498	M-H2O-H	-4.401917206	12	↓
P44	0009453	24.23	PE(20:5(5Z,8Z,11Z,14Z,17Z)/18:0)	C43H76NO8P	746.5125641	M-H2O-H	-0.587595458	2	↓

	P45	0009700	23.42	PE(22:6(4Z,7Z,10Z,13Z,16Z,19Z)/22:1(13Z))	C49H84NO8P	958.5646492	M-H	-1.731683401	3	↓
	P46	0009540	23.42	PE(22:1(13Z)/22:6(4Z,7Z,10Z,13Z,16Z,19Z))	C49H84NO8P	958.5646492	M-H	-1.731683401	3	↓
	P47	0009281	24.21	PE(20:1(11Z)/P-18:1(11Z))	C43H82NO7P	776.5556462	M+Na-2H	-2.530737761	3	↓
	P48	0009282	24.21	PE(20:1(11Z)/P-18:1(9Z))	C43H82NO7P	776.5556462	M+Na-2H	-2.530737762	3	↓
	P49	0009313	24.21	PE(20:2(11Z,14Z)/P-18:0)	C43H82NO7P	776.5556462	M+Na-2H	-2.530737762	3	↓
	P50	0009576	24.21	PE(22:2(13Z,16Z)/P-16:0)	C43H82NO7P	776.5556462	M+Na-2H	-2.530737762	3	↓
	P51	0006825	22.06	Tetrahydrofolyl-[Glu](2)	C24H30N8O9	593.212969	M-H	-1.796408905	6	↓
	P52	0001557	0.76	Riboflavin reduced	C15H16N4O6	367.1032806	M-H	-0.476009031	2	↑
	P53	0002925	21.07	Dihomo-gamma-linolenic acid	C20H34O2	305.2485881	M-H	-0.051570628	10	↑
	P54	0006791	6.40	Melibiitol	C12H24O11	343.1250448	M-H	1.335537469	2	↑
	P55	0000951	6.19	Taurochenodesoxycholic acid	C26H45NO6S	498.2872855	M-H	-4.40055667	12	↑
	P56	0000195	4.50	Inosine	C10H12N4O5	313.0782323	M+FA-H	-2.760455674	10	↑
	P57	0000663	4.33	Glucaric acid	C6H10O8	269.0482727	M+Hac-H	-1.64100219	3	↓
	P58	0000806	13.52	Myristic acid	C14H28O2	249.1842291	M+Na-2H	2.765671018	3	↑
	P59	0010381	10.09	LysoPC(15:0)	C23H48NO7P	480.3076509	M-H	-3.9728778	4	↓
	P60	0007855	9.91	LysoPA(18:1(9Z)/0:0)	C21H41O7P	457.2339923	M+Na-2H	0.765980412	2	↑
	P61	0000197	2.82	Indoleacetic acid	C10H9NO2	242.0478595	M-H	4.239021692	6	↑
	P62	0002925	10.41	Dihomo-gamma-linolenic acid	C20H34O2	305.2471548	M-H	-4.731588045	5	↑
ESI+	P63	0002995	21.26	12-Keto-tetrahydro-leukotriene B4	C20H34O4	371.2784315	M+K	-2.274127534	7	↓
	P64	0003208	13.49	Thromboxane	C20H40O	360.3239269	M+ACN+Na	0.813966482	13	↓
	P65	0004876	23.81	LacCer(d18:1/25:0)	C55H105NO13	1029.7939	M+ACN+H	1.530017275	7	↑
	P66	0012317	23.81	3-O-Sulfogalactosylceramide (d18:1/18:1(9Z))	C42H79NO11S	838.5610	M+Na	2.112738547	6	↑
	P67	0004949	23.50	Cer(d18:1/16:0)	C34H67NO3	502.4968369	M+H-2H2O	-2.61430559	2	↑

P68	0004837	23.72	Galabiosylceramide (d18:1/24:1(15Z))	C54H101NO13	994.7154136	M+Na	-1.131331338	21	↑
P69	0001547	6.05	Corticosterone	C21H30O4	347.2218899	M+H	0.589437874	13	↓
P70	0004096	3.75	5-Methoxyindoleacetate	C11H11NO3	250.0450217	M+Na	0.060563635	4	↑
P71	0000122	0.71	D-Glucose	C6H12O6	203.0522236	M+H-H2O	-2.138878061	8	↑
P72	0006547	20.12	Stearidonic acid	C18H28O2	259.2059895	M+H	1.258637786	4	↑
P73	0000175	0.66	Inosinic acid	C10H13N4O8P	355.0578571	M+2Na-H	0.034110709	5	↓
P74	0005358	23.80	TG(16:0/16:0/20:0)	C55H106O6	907.7710875	M+Na	1.137490066	7	↓
P75	0005389	23.80	TG(16:0/18:2(9Z,12Z)/20:1(11Z))	C57H104O6	907.7710875	M+H-2H2O	-1.609728224	7	↓
P76	0001070	23.57	Octanoyl-CoA	C29H50N7O17P3S	858.2050251	M+H	-0.891124617	4	↑
P77	0009379	23.57	PE(20:3(8Z,11Z,14Z)/P-18:0)	C43H80NO7P	753.5874621	M+Na	-0.867300072	9	↑
P78	0009346	23.57	PE(20:3(5Z,8Z,11Z)/P-18:0)	C43H80NO7P	753.5874621	M+H	-0.867300072	9	↑
P79	0009314	23.57	PE(20:2(11Z,14Z)/P-18:1(11Z))	C43H80NO7P	753.5874621	M+H	-0.867300072	9	↑
P80	0009315	23.57	PE(20:2(11Z,14Z)/P-18:1(9Z))	C43H80NO7P	753.5874621	M+2Na-H	-0.867300072	9	↑
P81	0004041	0.70	L-Allothreonine	C4H9NO3	164.0293778	M+H	-0.254434235	18	↓
P82	0000883	0.71	L-Valine	C5H11NO2	118.0859574	M+Na	-2.542403153	21	↓
P83	0006857	0.72	Dextrin	C18H32O16	527.1581486	M+H	-0.212347364	16	↓
P84	0006519	23.22	Stearidonoyl CoA	C39H62N7O17P3S	1026.318768	M+H	-2.031738961	16	-
P85	0000290	23.69	Uridine diphosphate-N-acetylglucosamine	C17H27N3O17P2	649.124109	M+NH4	-0.181965764	17	↓
P86	0004305	12.89	Farnesol	C15H26O	240.2322209	M+Na	0.134490285	12	↑
P87	0012453	14.99	3beta-Hydroxy-5-cholestenoic acid	C27H44O3	417.3362571	M+Na	-0.155186646	27	↑
P88	0011154	14.09	LysoPA(P-16:0/0:0)	C19H39O6P	417.3362571	M+H-2H2O	-0.897790707	18	↓
P89	0003141	13.85	Retinoyl b-glucuronide	C26H36O8	441.2252953	M+H-2H2O	-3.926386828	20	↓
P90	0010318	4.99	Pregnanediol 3-O-glucuronide	C27H44O8	535.2693022	M+Na	-1.993791678	10	↑
P91	0011105	6.35	5-Acetylamino-6-formylamino-3-	C12H21NO4S2	290.0870709	M+ACN+Na	-2.738468232	23	↑

			methyluracil						
P92	0011105	6.35	5-Acetylamino-6-formylamino-3-methyluracil	C8H10N4O4	290.0870709	M+ACN+Na	4.84959473	23	↑
P93	0008652	20.77	PC(22:4(7Z,10Z,13Z,16Z)/P-16:0)	C46H84NO7P	838.5584053	M+Na	-1.021520748	7	↑
P94	0008456	20.77	PC(20:4(5Z,8Z,11Z,14Z)/P-18:0)	C46H84NO7P	838.5584053	M+Na	-1.021520748	7	↑
P95	0001409	0.87	dUMP	C9H13N2O8P	353.0119925	M+Na	-0.404122646	8	↓
P96	0009710	21.80	PE(22:6(4Z,7Z,10Z,13Z,16Z,19Z)/P-18:1(11Z))	C45H76NO7P	818.5098938	M+Na	3.604809015	15	↑
P96	0009711	21.80	PE(22:6(4Z,7Z,10Z,13Z,16Z,19Z)/P-18:1(9Z))	C45H76NO7P	818.5098938	M+Na	3.604809015	15	↑
P97	0001496	21.38	7alpha-Hydroxycholesterol	C27H46O2	243.2104078	M+H	-1.148116411	3	↓
P98	0006228	18.39	24-Hydroxycalcitriol	C27H44O4	415.3185396	M+H	-4.931676159	7	↑
P99	0000827	17.84	Stearic acid	C18H36O2	285.2797433	M+Na	3.29424837	3	↑
P101	0001227	1.17	5-Thymidylic acid	C10H15N2O8P	401.0761598	M+H	-4.279794928	13	↑
P102	0001517	23.53	AICAR	C9H15N4O8P	371.1018086	M+H	-0.286035627	23	↓
P103	0000254	8.00	Succinic acid	C4H6O4	237.0586719	M+H	0.292131609	9	↑
P104	0006556	1.01	L-4-Hydroxyglutamate semialdehyde	C5H9NO4	136.0717	M+H	-2.291133552	5	↓
P105	0007942	23.13	PC(15:0/18:3(9Z,12Z,15Z))	C41H76NO8P	786.4966373	M+H	3.504142032	7	↑
P106	0000570	11.75	Coproporphyrin III	C36H38N4O8	619.2576422	M+H-2H2O	3.868397252	3	↓
P107	0007850	10.88	LysoPA(0:0/18:0)	C21H43O7P	502.2929364	M+H	-4.885840007	18	↑
P108	0000732	9.48	Hydroxykynurenine	C10H12N2O4	285.1440173	M+H	2.656410522	5	↑
P109	0005779	21.80	PE(P-18:0/20:4(5Z,8Z,11Z,14Z))	C43H78NO7P	751.5695842	M+H	-3.92743375	12	↑
