# Supplementary material for: Pueraria thomsonii Radix Water Extract Alleviate Type 2 Diabetes Mellitus in db/db Mice through Comprehensive Regulation of Metabolism and Gut Microbiota
Source: Molecules. 2023 Nov 7;28(22):7471. doi: 10.3390/molecules28227471 (PMC10673130; doi:10.3390/molecules28227471)
Supplement: Supplementary file 1 [file molecules-28-07471-s001.zip › Supplementary Table S3.pdf]

Supplementary Table S3.Differential metabolites for PTR callbacks

Modle	Name	Normal	Model	Up/down	PTR	Up/down	Metformin	Up/down
ESI-	13-L-Hydroperoxylinoleic acid	5.23±2.75	117.8±70.87	↑	15.26±21.40	↓	24.54±29.46	↓
	13-L-Hydroperoxylinoleic acid	0.51±0.50	38.89±27.04	↑	4.89±7.15	↓	25.06±52.20	↓
	11,12-Epoxyeicosatrienoic acid	15.99±7.74	49.73±13.08	↑	20.01±13.46	↓	7.66±5.88	↓
	5-HETE	15.99±7.74	49.73±13.08	↑	20.01±13.46	↓	7.66±5.88	↓
	Leukotriene D4	16.42±7.20	84.76±37.20	↑	32.76±38.05	↓	40.52±35.08	↓
	Prostaglandin H3	1.51±1.41	227.25±126.75	↑	30.71±50.87	↓	138.91±252.25	↓
	11,12-Epoxyeicosatrienoic acid	1.96±2.91	79.06±97.48	↑	9.31±12.73	↓	33.47±53.63	↓
	5a-Pregnane-3,20-dione	419.94±110.86	1421.76±580.48	↑	885.82±519.57	↓	654.02±513.41	↓
	2-Methoxy-estradiol-17b 3-glucuronide	51.17±16.50	180.9±58.08	↑	78.79±66.60	↓	89.81±69.88	↓
	11b,21-Dihydroxy-3,20-oxo-5b-pregnan-18-al	9.27±8.25	494.54±478.32	↑	70.37±99.37	↓	472.08±790.00	↓
	Estrone	6.35±2.49	41.93±17.60	↑	15.82±18.54	↓	50.38±94.13	↑
	5-L-Glutamyl-taurine	303.25±81.12	14.06±8.54	↓	188.23±398.96	↑	10.53±5.81	↓
	5-L-Glutamyl-taurine	215.19±65.25	36.6±20.49	↓	199.43±333.91	↑	22.44±12.34	↓
	Oxidized glutathione	4.14±5.43	37.54±23.34	↑	10.67±16.19	↓	4.13±6.99	↓
	Myristic acid	49.01±13.99	115.44±29.02	↑	77.58±35.44	↓	53.59±22.00	↓
	Chitobiose	47.32±18.41	162.84±38.96	↑	75.88±53.87	↓	48.67±43.82	↓

	Stearidonic acid	1.83±3.33	143.68±98.35	↑	31.95±46.10	↓	8.88±18.96	↓
	2,3-Diphosphoglyceric acid	52.06±17.34	128.62±29.17	↑	121.09±41.16	↓	93.17±33.69	↓
	Deoxyguanosine	50.19±20.83	139.25±31.29	↑	119.56±49.32	↓	94.47±39.78	↓
	Glucaric acid	43.27±19.25	4.04±4.20	↓	7.71±9.82	↑	2.19±2.04	↓
	Myristic acid	109.84±27.55	468.79±144.10	↑	222.72±103.53	↓	128.76±71.83	↓
	Dihomo-gamma-linolenic acid	92.62±28.55	193.39±72.82	↑	157.38±81.43	↓	133.39±109.52	↓
ESI+	12-Keto-tetrahydro-leukotriene B4	1048.74±973.85	395.47±129.55	↓	530.97±134.04	↑	465.45±143.25	↑
	Inosinic acid	566.82±272.11	192.92±126.99	↑	305.71±118.47	↑	174.12±137.58	↓
	LacCer(d18:1/25:0)	112.72±110.16	1676.13±1378.91	↑	811.7±593.07	↓	889.08±635.88	↓
	3-O-Sulfogalactosylceramide (d18:1/18:1(9Z))	24082.3±17753.64	68158.93±57739.78	↑	40884.38±23442.19	↓	37987.34±23998.72	↓
	Corticosterone	611.76±703.62	2536.35±830.51	↑	2073.39±576.52	↓	3278.3±650.40	↑
	5-Methoxyindoleacetate	209.06±41.23	493.89±120.13	↑	346.96±104.56	↓	715.01±262.34	↑
	D-Glucose	601.99±189.28	3014.81±953	↑	2211.98±676.49	↓	969.77±855.22	↓
	Octanoyl-CoA	136.04±289.67	525.94±449.6	↑	373.51±590.75	↓	1748.45±4602.18	↑
	PE(20:3(8Z,11Z,14Z)/P-18:0)	1464.82±912.57	4857.29±3210.04	↑	3287.19±2746.80	↓	4517.88±4230.66	↓
	PE(20:3(5Z,8Z,11Z)/P-18:0)	1464.82±912.57	4857.29±3210.04	↑	3287.19±2746.80	↓	4517.88±4230.66	↓
	PE(20:2(11Z,14Z)/P-18:1(11Z))	1464.82±912.57	4857.29±3210.04	↑	3287.19±2746.80	↓	4517.88±4230.66	↓
	PE(20:2(11Z,14Z)/P-18:1(9Z))	1464.82±912.57	4857.29±3210.04	↑	3287.19±2746.80	↓	4517.88±4230.66	↓

L-Allothreonine	231.76±53.49	36.28±10.82	↓	50.96±10.34	↑	80.66±26.25	↑
L-Valine	490.05±171.81	98.10±38.54	↓	108.54±35.96	↑	64.24±26.57	↓
3beta-Hydroxy-5-cholestenoic acid	10.61±12.32	552.16±255.49	↑	323.88±128.62	↓	488.98±187.49	↓
LysoPA(P-16:0/0:0)	1887.12±335.93	798.35±135	↓	972.99±122	↑	567.32±152.96	↓
Retinoyl b-glucuronide	451.78±91.88	201.27±55.26	↓	205.83±28.27	↑	206.01±65.41	↑
Pregnanediol 3-O-glucuronide	1.16±1.52	596.71±1494.4	↑	435.65±950.30	↓	160.17±501.95	↓
5-Acetylamino-6-formylamino-3-methyluracil	25.90±10.73	396.87±711.02	↑	27.97±4.55	↓	98.70±166.11	↓
5-Acetylamino-6-formylamino-3-methyluracil	25.90±10.73	396.87±711.02	↑	27.97±4.55	↓	98.70±166.11	↓
PC(22:4(7Z,10Z,13Z,16Z)/P-16:0)	14591.12±15077.24	57314.16±73203.13	↑	26963.18±24312.5	↓	30779.08±31862.64	↓
PC(20:4(5Z,8Z,11Z,14Z)/P-18:0)	14591.12±15077.24	57314.16±73203.13	↑	26963.18±24312.50	↓	30779.08±31862.64	↓
dUMP	324.58±123.12	103.79±29.58	↓	184.69±37.50	↑	130.73±32.29	↑
7alpha-Hydroxycholesterol	405.93±336.97	127.24±17.68	↓	130.11±19.53	↑	116.53±16.78	↓
24-Hydroxycalcitriol	192.29±72.50	441.69±111.43	↑	401.48±101.01	↓	740.14±180.84	↑
5-Thymidylic acid	497.61±544.12	2122.12±1346.42	↑	1677.69±988.40	↓	1780.46±524.14	↓
Succinic acid	157.26±37.15	608.65±330.25	↑	528.56±258.75	↓	330.88±157.75	↓
Hydroxykynurenine	111.64±31.68	316.89±71.56	↑	213.53±53.80	↓	180.43±83.52	↓
9,10-EOA	118.33±62.50	462.34±103.71	↑	28.93±81.12	↓	66.14±83.12	↓
12,13-EpOME	83.80±20.47	169.71±59.65	↑	44.00±48.94	↓	21.05±25.15	↓

Spermine	83.80±20.47	169.71±59.65	↑	44.00±48.94	↓	21.05±25.15	↓
SM(d18:1/26:1(17Z))	7.72±9.63	40.34±19.40	↑	4.56±7.20	↓	4.00±6.65	↓
3beta-Hydroxypregn-5-en-20-one sulfate	18.02±6.83	78.37±38.48	↑	1.30±2.23	↓	50.05±103.88	↓
Dehydroepiandrosterone sulfate	24.16±9.49	74.72±26.30	↑	18.96±16.24	↓	17.46±12.49	↓
Cortisol	42.05±32.20	394.79±497.67	↑	39.61±64.27	↓	116.49±242.76	↓
Cortisol	8.07±8.55	103.66±146.81	↑	5.42±13.13	↓	25.99±56.50	↓
11b,21-Dihydroxy-3,20-oxo-5b-pregnan-18-al	171.58±41.18	345.22±146.90	↑	74.19±92.74	↓	86.54±108.68	↓
Taurocholic acid	2715.54±987.72	8668.35±2877.01	↑	701.56±2109.59	↓	233.06±531.20	↓
5-Methoxyindoleacetate	299.31±90.20	769.36±312.72	↑	30.51±51.14	↓	182.68±372.96	↓
LysoPC(20:4(8Z,11Z,14Z,17Z)/0:0)	22.00±9.33	55.07±20.99	↑	7.69±12.84	↓	15.50±26.03	↓
Indoleacetaldehyde	61.75±22.08	173.26±79.66	↑	8.87±11.35	↓	37.88±78.20	↓
Orotidylic acid	39.57±15.98	99.37±41.60	↑	18.57±19.24	↓	27.95±40.34	↓
7,8-Dihydro-7-hydroxy-8-S-glutathionyl-benzo[a]pyrene	46.17±17.39	115.08±35.02	↑	45.15±35.96	↓	26.31±23.61	↓
Tetrahydrofolyl-[Glu](2)	222.00±144.65	50.16±22.42	↓	525.67±333.74	↑	189.22±270.73	↑
Riboflavin reduced	151.59±88.03	446.37±371.84	↑	23.07±26.04	↓	61.37±59.39	↓
Dihomo-gamma-linolenic acid	2179.79±443.66	4525.65±1071.58	↑	1809.02±1706.24	↓	2654.01±1489.12	↓
Melibiitol	23.57±8.00	59.05±20.91	↑	13.33±21.05	↓	14.57±22.27	↓
Taurochenodesoxycholic acid	93.71±35.81	662.2±939.04	↑	54.44±90.91	↓	23.87±63.64	↓

Inosine	45.56±9.49	100.34±54.31	↑	6.70±13.05	↓	23.83±48.44	↓
LysoPA(18:1(9Z)/0:0)	33.34±13.90	81.2±21.10	↑	14.86±20.26	↓	23.48±33.83	↓
Indoleacetic acid	27.02±8.03	85.85±40.24	↑	12.86±21.31	↓	113.38±233.99	↑
