# Supplementary material for: Pueraria thomsonii Radix Water Extract Alleviate Type 2 Diabetes Mellitus in db/db Mice through Comprehensive Regulation of Metabolism and Gut Microbiota
Source: Molecules. 2023 Nov 7;28(22):7471. doi: 10.3390/molecules28227471 (PMC10673130; doi:10.3390/molecules28227471)
Supplement: Supplementary file 1 [file molecules-28-07471-s001.zip › Supplementary Table S4.pdf]

Supplementary Table S4.Differential metabolites for Metformin callbacks

Modle	Name	Normal	Model	Metformin	Up/down
ESI-	13-L-Hydroperoxylinoleic acid	5.23±2.75	117.8±70.87	24.54±29.46	↓
	13-L-Hydroperoxylinoleic acid	0.51±0.50	38.89±27.04	25.06±52.20	↓
	15H-11,12-EETA	37.48±21.98	2.57±2.25	3.70±9.26	↑
	Leukotriene D4	16.42±7.20	84.76±37.20	40.52±35.08	↓
	Prostaglandin H3	1.51±1.41	227.25±126.75	138.91±252.25	↓
	11,12-Epoxyeicosatrienoic acid	1.96±2.91	79.06±97.48	33.47±53.63	↓
	5a-Pregnane-3,20-dione	419.94±110.86	1421.76±580.48	654.02±513.41	↓
	3beta-Hydroxypregn-5-en-20-one sulfate	18.02±6.83	78.37±38.48	50.05±103.88	↓
	2-Methoxy-estradiol-17b 3-glucuronide	51.17±16.50	180.9±58.08	89.81±69.88	↓
	Cortisol	42.05±32.20	394.79±497.67	116.49±242.76	↓
	11b,21-Dihydroxy-3,20-oxo-5b-pregnan-18-al	9.27±8.25	494.54±478.32	472.08±790.00	↓
	Cortisol	8.07±8.55	103.66±146.81	25.99±56.50	↓
	Myristic acid	49.01±13.99	115.44±29.02	53.59±22.00	↓
	2,3-Diphosphoglyceric acid	52.06±17.34	128.62±29.17	93.17±33.69	↓
	Deoxyguanosine	50.19±20.83	139.25±31.29	94.47±39.78	↓
	PE(18:2(9Z,12Z)/P-	853.17±690.86	108.02±85.89	123.19±173.78	↑

	18:1(11Z))				
	PE(18:2(9Z,12Z)/P-18:1(9Z))	853.17±690.86	108.02±85.89	123.19±173.78	↑
	PE(20:5(5Z,8Z,11Z,14Z,17Z)/18:0)	42.38±34.01	3.38±4.78	4.88±6.29	↑
	Tetrahydrofolyl-[Glu](2)	222.00±144.65	50.16±22.42	189.22±270.73	↑
	Dihomo-gamma-linolenic acid	2179.79±443.66	4525.65±1071.58	2654.01±1489.12	↓
	Myristic acid	109.84±27.55	468.79±144.10	128.76±71.83	↓
	Dihomo-gamma-linolenic acid	92.62±28.55	193.39±72.82	133.39±109.52	↓
ESI+	12-Keto-tetrahydro-leukotriene B4	1048.74±973.85	395.47±129.55	465.45±143.25	↑
	LacCer(d18:1/25:0)	112.72±110.16	1676.13±1378.91	889.08±635.88	↓
	3-O-Sulfogalactosylceramide (d18:1/18:1(9Z))	24082.3±17753.64	68158.93±57739.78	37987.34±23998.72	↓
	Cer(d18:1/16:0)	117.63±55.76	399.94±286.03	397.75±277.39	↓
	D-Glucose	601.99±189.28	3014.81±953	969.77±855.22	↓
	PE(20:3(8Z,11Z,14Z)/P-18:0)	1464.82±912.57	4857.29±3210.04	4517.88±4230.66	↓
	PE(20:3(5Z,8Z,11Z)/P-18:0)	1464.82±912.57	4857.29±3210.04	4517.88±4230.66	↓
	PE(20:2(11Z,14Z)/P-18:1(11Z))	1464.82±912.57	4857.29±3210.04	4517.88±4230.66	↓
	PE(20:2(11Z,14Z)/P-	1464.82±912.57	4857.29±3210.04	4517.88±4230.66	↓

18:1(9Z))				
L-Allothreonine	231.76±53.49	36.28±10.82	80.66±26.25	↑
Dextrin	455.29±127.05	32.62±21.97	63.12±49.13	↑
Farnesol	209.95±36.21	574.61±483.27	572.22±453.66	↓
3beta-Hydroxy-5-cholestenoic acid	10.61±12.32	552.16±255.49	488.98±187.49	↓
Retinoyl b-glucuronide	451.78±91.88	201.27±55.26	206.01±65.41	↑
Pregnanediol 3-O-glucuronide	1.16±1.52	596.71±1494.4	160.17±501.95	↓
5-Acetylamino-6-formylamino-3-methyluracil	25.90±10.73	396.87±711.02	98.70±166.11	↓
5-Acetylamino-6-formylamino-3-methyluracil	25.90±10.73	396.87±711.02	98.70±166.11	↓
PC(22:4(7Z,10Z,13Z,16Z)/P-16:0)	14591.12±15077.24	57314.16±73203.13	30779.08±31862.64	↓
PC(20:4(5Z,8Z,11Z,14Z)/P-18:0)	14591.12±15077.24	57314.16±73203.13	30779.08±31862.64	↓
dUMP	324.58±123.12	103.79±29.58	130.73±32.29	↑
5-Thymidylic acid	497.61±544.12	2122.12±1346.42	1780.46±524.14	↓
AICAR	5991.18±3895.68	2990.63±976.2	2331.56±452.19	↓
Succinic acid	157.26±37.15	608.65±330.25	330.88±157.75	↓
L-4-Hydroxyglutamate semialdehyde	365.55±79.29	124.77±26.37	151.44±53.75	↑
PC(15:0/18:3(9Z,12Z,15Z))	267.03±318.75	733±522.52	553.37±260.70	↓
LysoPA(0:0/18:0)	26.43±15.31	245.75±140.45	102.08±63.76	↓
Hydroxykynurenine	111.64±31.68	316.89±71.56	180.43±83.52	↓
