# Supplementary material for: Pueraria thomsonii Radix Water Extract Alleviate Type 2 Diabetes Mellitus in db/db Mice through Comprehensive Regulation of Metabolism and Gut Microbiota
Source: Molecules. 2023 Nov 7;28(22):7471. doi: 10.3390/molecules28227471 (PMC10673130; doi:10.3390/molecules28227471)
Supplement: Supplementary file 1 [file molecules-28-07471-s001.zip › Supplementary Table S5.pdf]

Supplementary Table S5. Analysis of PTR metabolic pathway enrichment

Pathway Name	Match Status	-log(p)	Holm p	FDR	Impact	Details
Taurine and hypotaurine metabolism	2/8	6.8401	5.7805E-6	2.964E-6	0.0	KEGG SMP
Primary bile acid biosynthesis	4/46	6.8291	5.7805E-6	2.964E-6	0.06417	KEGG SMP
Tryptophan metabolism	3/41	3.7534	0.0067052	0.0021579	0.0139	KEGG SMP
Riboflavin metabolism	1/4	3.666	0.0079842	0.0021579	0.0	KEGG SMP
Glutathione metabolism	2/28	3.3235	0.017091	0.0027412	0.02698	KEGG SMP
beta-Alanine metabolism	1/21	3.319	0.017091	0.0027412	0.05597	KEGG SMP
Arginine and proline metabolism	1/38	3.319	0.017091	0.0027412	0.0	KEGG SMP
Glycerolipid metabolism	1/16	2.7966	0.052718	0.0071	0.01246	KEGG SMP
Phosphatidylinositol signaling system	1/28	2.7966	0.052718	0.0071	0.00152	KEGG
Glycine, serine and threonine metabolism	1/34	2.5244	0.092684	0.011959	0.0	KEGG SMP
Purine metabolism	3/66	1.7577	0.52415	0.059558	0.13284	KEGG SMP
Metabolism of xenobiotics by cytochrome P450	1/64	1.7479	0.52415	0.059558	0.0	KEGG
Glycolysis / Gluconeogenesis	2/26	1.5236	0.83852	0.085352	0.04443	KEGG SMP SM P
Steroid hormone biosynthesis	7/77	1.4957	0.86229	0.085352	0.10964	KEGG
Ascorbate and aldarate	1/10	1.4948	0.86229	0.085352	0.0	KEGG

metabolism						
Galactose metabolism	1/27	1.1258	1.0	0.18714	0.03577	KEGG SMP
Caffeine metabolism	1/12	1.0081	1.0	0.23096	0.0	KEGG SMP
Sphingolipid metabolism	3/21	0.80391	1.0	0.34904	0.0	KEGG SMP
Folate biosynthesis	1/27	0.70755	1.0	0.35187	0.0	KEGG
Glycerophospholipid metabolism	3/36	0.69438	1.0	0.35187	0.33882	KEGG
Arachidonic acid metabolism	4/36	0.69396	1.0	0.35187	0.0424	KEGG SMP
Linoleic acid metabolism	3/5	0.69395	1.0	0.35187	0.0	KEGG
alpha-Linolenic acid metabolism	2/13	0.69395	1.0	0.35187	0.0	KEGG
Glycosylphosphatidylinositol (GPI)-anchor biosynthesis	1/14	0.65376	1.0	0.3699	0.00399	KEGG
Biosynthesis of unsaturated fatty acids	1/36	0.63577	1.0	0.37012	0.0	KEGG
Pyrimidine metabolism	3/39	0.4317	1.0	0.5538	0.26541	KEGG SMP
Steroid biosynthesis	1/42	0.42554	1.0	0.5538	0.0	KEGG SMP
Fatty acid biosynthesis	1/47	0.34339	1.0	0.5538	0.0	KEGG SMP
Fatty acid degradation	1/39	0.30867	1.0	0.5538	0.02355	KEGG SMP
Fatty acid elongation	1/39	0.30867	1.0	0.5538	0.01148	KEGG SMP
Valine, leucine and isoleucine degradation	1/40	0.29304	1.0	0.5538	0.0	KEGG SMP
Valine, leucine and isoleucine biosynthesis	1/8	0.29304	1.0	0.5538	0.0	KEGG

Pantothenate and CoA biosynthesis	1/19	0.29304	1.0	0.5538	0.0	KEGG SMP
Aminoacyl-tRNA biosynthesis	1/48	0.29304	1.0	0.5538	0.0	KEGG
Citrate cycle (TCA cycle)	1/20	0.27893	1.0	0.5538	0.03273	KEGG
Alanine, aspartate and glutamate metabolism	1/28	0.27893	1.0	0.5538	0.0	KEGG SMP
Propanoate metabolism	1/23	0.27893	1.0	0.5538	0.0	KEGG SMP
Butanoate metabolism	1/15	0.27893	1.0	0.5538	0.0	KEGG
Amino sugar and nucleotide sugar metabolism	1/37	0.21709	1.0	0.62216	0.0	KEGG SMP SMP
Retinol metabolism	1/16	0.092641	1.0	0.8079	0.0	KEGG SMP
