# Supplementary material for: Pueraria thomsonii Radix Water Extract Alleviate Type 2 Diabetes Mellitus in db/db Mice through Comprehensive Regulation of Metabolism and Gut Microbiota
Source: Molecules. 2023 Nov 7;28(22):7471. doi: 10.3390/molecules28227471 (PMC10673130; doi:10.3390/molecules28227471)
Supplement: Supplementary file 1 [file molecules-28-07471-s001.zip › Supplementary Table S6.pdf]

Supplementary Table S6. Analysis of Metformin metabolic pathway enrichment

Pathway Name	Match Status	-log(p)	Holm p	FDR	Impact	Details
Fatty acid biosynthesis	1/47	4.8527	3.6499E-4	2.4636E-4	0.0	KEGG SMP
Glycine, serine and threonine metabolism	1/34	4.6184	6.0186E-4	2.4636E-4	0.0	KEGG SMP
Glycolysis / Gluconeogenesis	2/26	4.5463	6.8222E-4	2.4636E-4	0.04443	KEGG SMP SMP
Biosynthesis of unsaturated fatty acids	1/36	2.6078	0.056741	0.016035	0.0	KEGG
Glycerolipid metabolism	1/16	2.178	0.14602	0.028761	0.01246	KEGG SMP
Phosphatidylinositol signaling system	1/28	2.178	0.14602	0.028761	0.00152	KEGG
Steroid hormone biosynthesis	4/77	1.8829	0.26189	0.048636	0.02841	KEGG
Citrate cycle (TCA cycle)	1/20	1.6393	0.43602	0.054241	0.03273	KEGG
Alanine, aspartate and glutamate metabolism	1/28	1.6393	0.43602	0.054241	0.0	KEGG SMP
Propanoate metabolism	1/23	1.6393	0.43602	0.054241	0.0	KEGG SMP
Butanoate metabolism	1/15	1.6393	0.43602	0.054241	0.0	KEGG
Purine metabolism	2/66	1.2166	0.91088	0.13157	0.07491	KEGG SMP
Starch and sucrose metabolism	1/15	1.1737	0.93857	0.13408	0.0	KEGG SMP
Folate biosynthesis	1/27	1.0398	1.0	0.16946	0.0	KEGG
Sphingolipid metabolism	3/21	0.86849	1.0	0.23463	0.26978	KEGG SMP
Arginine and proline	1/38	0.83888	1.0	0.23549	0.0	KEGG SMP

metabolism						
Caffeine metabolism	1/12	0.68523	1.0	0.31571	0.0	KEGG SMP
Glycerophospholipid metabolism	3/36	0.61614	1.0	0.34959	0.33882	KEGG
Linoleic acid metabolism	2/5	0.55501	1.0	0.38125	0.0	KEGG
Arachidonic acid metabolism	5/36	0.52181	1.0	0.39096	0.0636	KEGG SMP
alpha-Linolenic acid metabolism	1/13	0.47896	1.0	0.41096	0.0	KEGG
Pyrimidine metabolism	2/39	0.34187	1.0	0.53787	0.19576	KEGG SMP
Primary bile acid biosynthesis	1/46	0.28245	1.0	0.58992	0.0	KEGG SMP
Glycosylphosphatidylinositol (GPI)-anchor biosynthesis	1/14	0.10069	1.0	0.85916	0.00399	KEGG
Retinol metabolism	1/16	0.067713	1.0	0.88986	0.0	KEGG SMP
N-Glycan biosynthesis	1/41	0.0040888	1.0	0.99063	0.0	KEGG
